# The bacterial stress response polymerase DinB tolerates sugar modifications and preferentially incorporates arabinosyl nucleotides

**DOI:** 10.1039/d5cb00100e

**Published:** 2025-09-01

**Authors:** Christina M. Hurley, Jeffrey M. Kubiak, Michael B. Cory, Jared B. Parker, Christian E. Loo, Laura C. Wang, Rahul M. Kohli

**Affiliations:** a Graduate Group in Biochemistry, Biophysics, and Chemical Biology, Perelman School of Medicine, University of Pennsylvania Philadelphia PA USA; b Graduate Group in Cell and Molecular Biology, Perelman School of Medicine, University of Pennsylvania Philadelphia PA USA; c Department of Medicine, Perelman School of Medicine, University of Pennsylvania Philadelphia PA USA; d Department of Biochemistry and Biophysics, Perelman School of Medicine, University of Pennsylvania Philadelphia PA USA rkohli@pennmedicine.upenn.edu

## Abstract

The bacterial DNA damage (SOS) response promotes DNA repair, DNA damage tolerance, and survival in the setting of genotoxic stress, including stress induced by antibiotics. In *E. coli*, translesion DNA synthesis can be fulfilled by Y-family DNA polymerases, including DNA polymerase IV (DinB). DinB features a more open active site and lacks proofreading ability, promoting error-prone replication. While DinB is known to tolerate damaged nucleobases like 8-oxo-guanine (8-oxoG), its ability to accommodate sugar-modified nucleotides has been underexplored, a question of importance given that such analogs are commonly used to inhibit viral and other polymerases. To explore DinB's selectivity, we screened a variety of sugar-modified noncanonical nucleotide triphosphates (nNTPs) and determined that DinB is intolerant of most 3′-modifications but can incorporate a subset of 2′-modifications. In particular, arabinosyl nucleotide triphosphates (araNTPs) showed efficient incorporation and limited extension. Furthermore, araNTPs can effectively compete with natural nucleotide triphosphates leading to stalled replication by DinB. We show that this tolerance extends to combined nucleobase and sugar modifications, with preferred misincorporation of 2′-fluoroarabinosyl-8-oxo-GTP opposite A more than C. Overall, our work highlights the potential for exploiting substrate promiscuity to target DinB and, thereby, slow bacterial adaptation to antibiotics.

## Abstract 2

The promiscuous bacterial DNA polymerase DinB tolerates a subset of sugar-modified NTPs, offering a strategy for slowing escape from antibiotics.

## Graphical abstract

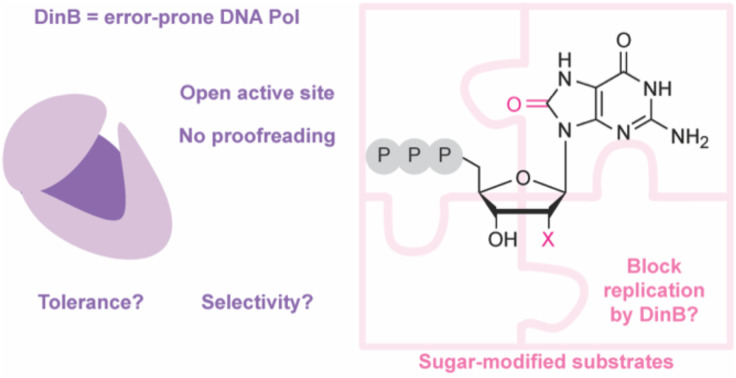

## Introduction

**Fig. 1:**
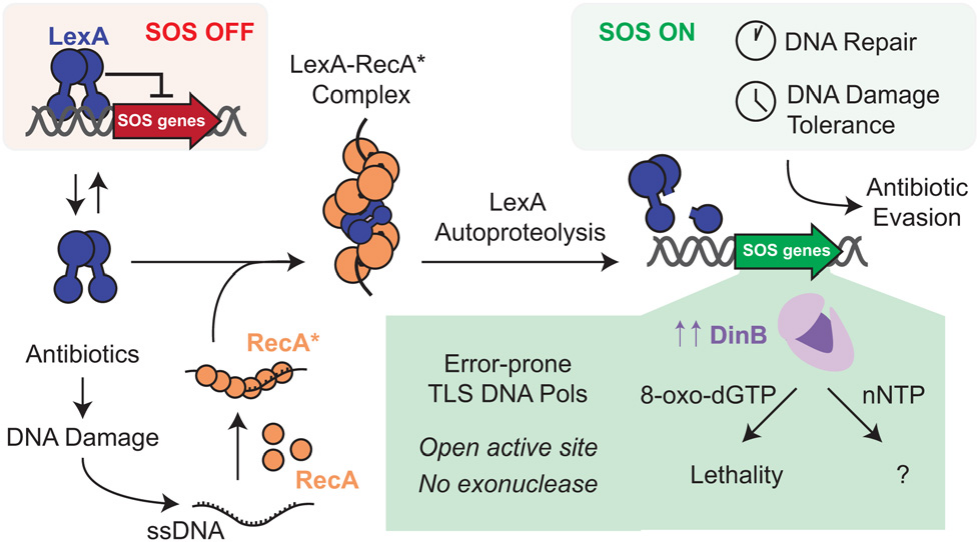
DinB as an SOS-induced translesion synthesis DNA polymerase. DNA damage, including that from antibiotics, leads to activation of the SOS response, which is regulated by LexA (blue) and RecA (orange). In the absence of DNA damage, LexA is a transcriptional repressor that suppresses SOS gene induction. In the presence of DNA damage, RecA polymerizes on ssDNA forming activated RecA (RecA*), which stimulates autoproteolysis of LexA. LexA cleavage leads to chronological induction of SOS response genes, including the error-prone translesion synthesis DNA polymerase DinB (purple). DinB mediates DNA damage tolerance, which can lead to antibiotic evasion. Given its open active site and lack of proofreading, DinB is known to tolerate nucleobase modifications; however, its tolerance for sugar-modified nucleotides is underexplored.

The SOS response is the conserved bacterial DNA damage response, a stress-induced pathway strongly implicated in tolerance to and escape from antibiotics.^1,2^ In the SOS response, DNA damage leads to the accumulation of single-stranded DNA, which serves as a template for the oligomerization and activation of RecA. Activated RecA (RecA*) promotes the subsequent cleavage of the transcriptional repressor, LexA (Fig. 1).^3^ Upon LexA cleavage, an array of SOS response genes are induced in a cascading fashion.^4,5^ While early effectors typically mediate high-fidelity repair, lower-fidelity processes predominate at later stages when DNA damage is too extensive to correct accurately.^5,6^ Key mediators of the latter processes are the specialized group of translesion synthesis (TLS) DNA polymerases that can replicate over damaged templates, albeit at the cost of fidelity.^7–9^ As predicted given these roles, deletion of TLS polymerases has shown that these effectors can contribute both to survival in the setting of antibiotic stress and the acquisition of antibiotic resistance.^10–13^

In *E. coli*, SOS-induced TLS polymerases include the Y-family polymerases, DNA Pol V and DNA Pol IV (DinB), the latter of which is induced at least 10-fold as part of the SOS response.^14–16^ While its canonical role relates to replication over damaged templates,^8,9^ DinB and its bacterial homologs can also misincorporate canonical bases or tolerate nucleobase variations in incoming deoxynucleotide triphosphates (dNTPs).^17,18^ For example, DinB readily misincorporates the 8-oxoguanine damaged form of dGTP (8-oxo-dGTP) opposite A using Hoogsteen base pairing. This activity can be lethal to *E. coli* as DNA repair mechanisms are triggered in a way that compromises genomic integrity.^19^ The tolerance of DinB to template and base modifications is attributable to at least two features: its more open active site and lack of proofreading activity.^20^ Structural and biochemical studies have shown that the active site is marked by an N-terminal catalytic core with a fingers domain that is smaller and less flexible than those of high-fidelity replicative polymerases, with less dynamic motions upon engagement with an incoming nucleotide (Fig. S1A).^21,22^ The pro-mutagenic nature of DinB is further amplified by the absence of an exonuclease domain, which allows for extension past non-optimal base pairs and bypass of genomic lesions.

Notably, nonnatural nucleotides have found utility as anti-cancer and anti-infective agents, with targeting of the HSV DNA polymerase by acyclovir and nucleoside reverse transcriptase inhibitors targeting HIV as compelling examples.^23,24^ Given that many of these approaches rely on exploiting polymerase tolerance for specific sugar-modified NTPs, we considered whether DinB might show similar promiscuity that could support future efforts to develop inhibitors to slow acquired antibiotic resistance (Fig. 1). Prior work has established that DinB, like many DNA polymerases, can exclude ribonucleotide triphosphates (rNTPs).^25–27^ However, as the broader selectivity for sugar-modified NTPs has been underexplored, we aimed to systematically examine DinB's ability to utilize such substrates. Our analysis reveals that arabinosyl nucleotide triphosphates (araNTPs) can be readily incorporated by DinB and stall further elongation, even competing effectively with natural dNTPs. We further demonstrate synergy in nucleobase and sugar modifications, with 2′-fluoroarabinosyl-8-oxo-GTP (8-oxo-2′-F-araGTP) as an analog that is efficiently misincorporated opposite A by DinB. Overall, our biochemical analysis reveals insights into DinB substrate selectivity and highlights potential avenues for targeting these SOS-regulated polymerases as a strategy for slowing acquired antibiotic resistance.

## Results and discussion

### DinB tolerates a subset of 2′-sugar modifications

**Fig. 2:**
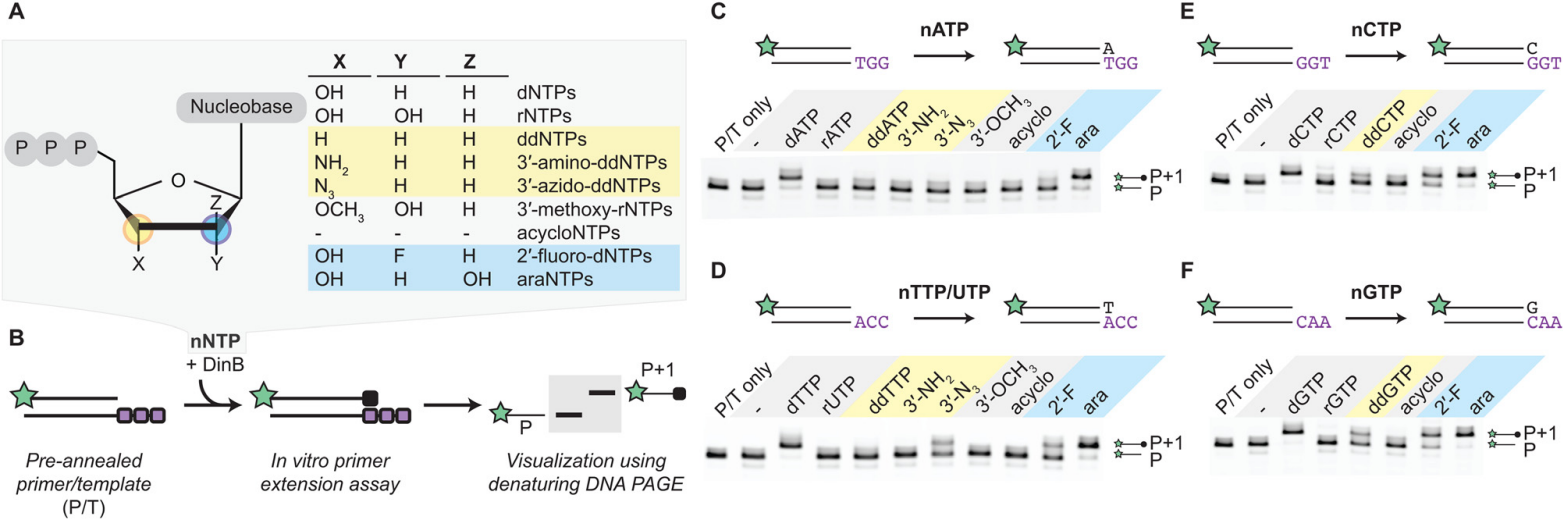
DinB efficiently incorporates arabinosyl nucleotides (araNTPs). (A) Natural dNTPs, rNTPs, and various sugar-modified noncanonical NTPs (nNTPs) evaluated as candidates. (B) Candidates are examined as substrates for DinB using a single-nucleotide extension assay (50 nM primer/template, 250 nM DinB, 50 μM nNTP). (C)–(F) Single nucleotide incorporation with (C) nATP, (D) nTTP/UTP, (E) nCTP, and (F) nGTP analogs are shown, with quantification in Fig. S7. For (D), all T/U analogs have a T nucleobase other than rUTP, 3′-methoxy-rUTP, and araUTP. Extension reactions were quantified in duplicate (Fig. S3), with no efficient extension defined as detection of <10% P + 1 band under these reaction conditions.

Like most DNA polymerases, DinB is known to exclude rNTPs due to a steric gate residue, F13 in the case of *E. coli* DinB, that probes the presence of the ribosyl 2′-*R*-hydroxyl (Fig. S1B).^25–27^ While the mechanism for rNTP exclusion has been explored, we considered whether other sugar-modified, noncanonical nucleotide triphosphates (nNTPs) might be tolerated given the distinctive active site and absence of proofreading activity. To this end, we purified *E. coli* DinB to near homogeneity (Fig. S1C–E). We aimed to study its ability to incorporate diverse nucleotides with modifications at the 2′- and 3′-positions, as well as acyclo analogs (acyNTPs) (Fig. 2(A)). To detect individual incorporation events with these nNTPs, we used a fluorescence-based, *in vitro* primer extension assay. DNA substrates (Fig. S2A–D) were designed so that a 13-nucleotide FAM-labeled primer is annealed to a complementary oligonucleotide leaving a three-nucleotide overhang to template the incorporation of incoming nucleotide triphosphates. This pre-annealed primer/template (P/T) is then incubated with DinB and either the dNTP or nNTP of interest. Once the reaction is complete, the fluorescent primer (P) and extended primer (P + 1) can be visualized on a denaturing gel (Fig. 2(B)). Notably, a small amount of P-1 product could also be detected on incubation of P/T with DinB, likely attributable to trace co-purifying exonuclease (Fig. S2E).

For our initially broad screen of nNTPs, we designed the templates to incorporate only one cognate nNTP and evaluated under substrate conditions where selectivity could be readily parsed between known favored (dNTP) and disfavored (rNTP) substrates. Starting with our adenine substrate series and a template oligonucleotide with a 3′-TGG-5′ overhang, we observed the anticipated efficient P to P + 1 extension with dATP (Fig. 2(C)). No efficient extension was observed using rATP after the incubation, where lack of efficient extension is defined as <10% P + 1 product detected upon quantification under these fixed reaction conditions (Fig. S3). Looking at various 3′-modified analogs, we observed that dideoxy (ddATP), 3′-amino, 3′-azido, and 3′-methoxy analogs also show no efficient extension under these conditions, highlighting a role for the 3′-OH in the stabilization of the incoming nucleotide. This observation is further established by the inability of DinB to efficiently utilize acyATP as a substrate for primer extension. Moving to 2′-variants, we observed partial incorporation using 2′-fluoro-dATP, suggesting that the smaller 2′-*R* moiety could be tolerated better than the ribosyl 2′-*R*-OH. We were most intrigued, however, by observations with reversion of the 2′-substituent stereochemistry in araATP relative to rATP. We found that extension with arabinosyl-ATP (araATP) was comparable to that of dATP. This finding suggests that the steric gate residue of DinB does not exclude the 2′-*S* moiety, potentially due to the propensity for araNTPs to adopt dNTP-like sugar pucker conformations.^28^

To understand the generalizability of these results, we extended our analysis to include available sugar-modified NTP analogs of T/U, C, and G (Table S1). For T/U analogs, we observed a pattern generally consistent with A analogs. The exception was 3′-azido-ddTTP, which showed inefficient but detectable incorporation (Fig. 2(D)). For C and G analogs, we observed some increased tolerance. While acyNTPs were again not efficiently incorporated, partial incorporation of ddCTP and ddGTP was observed, along with improved incorporation of 2′-fluoro-dCTP and 2′-fluoro-dGTP (Fig. 2(E) and (F)). This contrasts with the behavior of A/T analogs and may reflect the enhanced H-bonding capacity of G/C analog base pairs. Most importantly, however, we again observed that araNTPs were efficiently utilized as substrates for incorporation, with araUTP, araCTP, and araGTP all demonstrating near-complete conversion to the P + 1 product under these discriminating reaction conditions. Overall, these results suggested that there might be flexibility in tolerance for nucleotide modifications, with araNTPs being the most readily incorporated analogs.

### DinB does not efficiently extend after araNTP incorporation

**Fig. 3:**
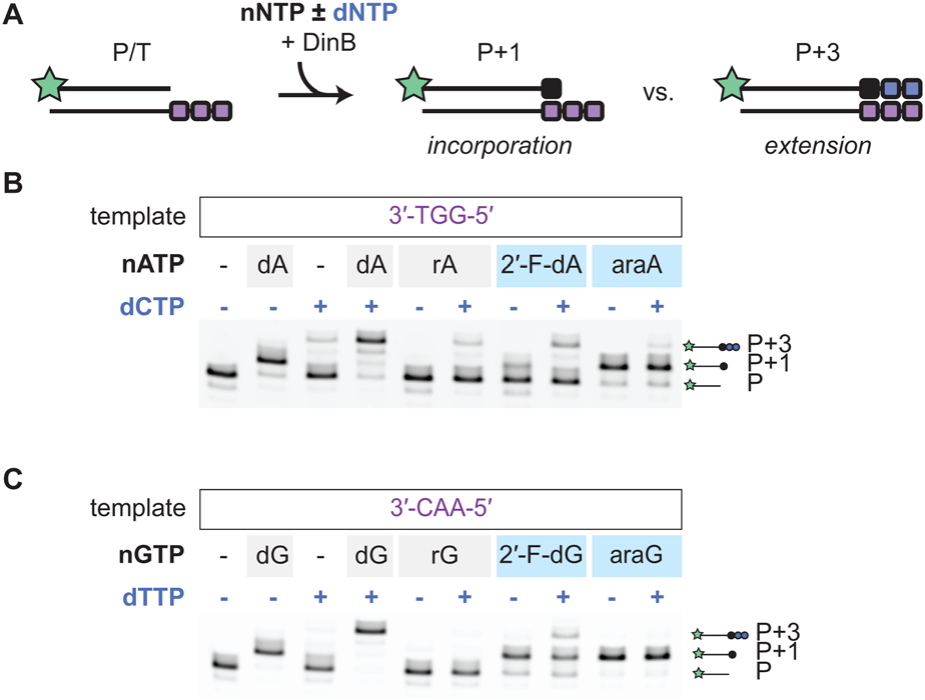
AraNTP incorporation leads to inefficient extension. (A) Assay schematic for incorporation and extension (50 nM primer/template, 250 nM DinB, 50 μM each nNTP ± dNTP). (B) and (C) Shown are incorporation and extension with (B) nATP ± dCTP, or (C) dGTP ± dTTP, with quantification provided in Fig. S8.

The ability to block (*e.g.*, AZT, ddNTPs) or slow extension (*e.g.*, entecavir, cytarabine) after incorporation has been a successful strategy in antiviral and anticancer approaches.^29^ Having explored incorporation by DinB, we next asked whether a subset of sugar-modified NTPs also impacted primer extension. These primer extension assays were similar to the single-incorporation screening assays, except that the nNTP of choice was co-incubated with the dNTP required to extend the primer beyond P + 1 (Fig. 3(A)). To start, we again focused on the A analogs with template containing the 3′-TGG-5′ overhang. While dATP alone lead to formation of the P + 1 product, this product is extended to form the P + 3 product in the presence of dCTP (Fig. 3(B)). Notably, dCTP alone can result in some detectable P + 3 product, attributable to misincorporation, followed by extension with the cognate dNTP. The pattern with rATP and dCTP is similar to that of dCTP alone. With 2′-F-dATP, a limited P + 1 extension product is detected in the absence of dCTP and subsequent extension of this product in the presence of dCTP. With araATP, we observed a distinct pattern from the other analogs tested. The araATP is efficiently incorporated to make the P + 1 product, but the P + 3 extension product is not readily observed over background levels with dCTP alone.

We next extended our analysis to the other sugar-modified NTP analogs of T/U (Fig. S4A), C (Fig. S4B), and G (Fig. 3(C)). For these analogs, we noted a similar pattern to that observed with sugar-modified A analogs with a few exceptions. While rUTPs are not efficiently incorporated and thus minimally extended, some extension could again be observed after incorporation of the 2′-F-dNTPs in the presence of cognate dNTP for extension, especially for 2′-F-dCTP. Most notably, araUTP, araCTP, and araGTP showed patterns consistent with araATP. Despite the presence of the cognate dNTP necessary for extension to P + 2 or P + 3, there is limited extension beyond generation of the P + 1 product. To more comprehensively analyze stalling of extension, we allowed extension with araGTP to proceed to efficiently generate the P + 1 product and then subsequently added additional dTTP to reach a 10-fold higher concentration (500 μM). Even under these conditions, we again did not observe significant extension past the P + 1 product (Fig. S4C). Together, these results show that DinB does not efficiently catalyze extension once araNTPs have been incorporated, suggesting that araNTPs could potentially stall DinB-dependent DNA replication.

### araNTPs can compete with their natural counterparts

**Fig. 4:**
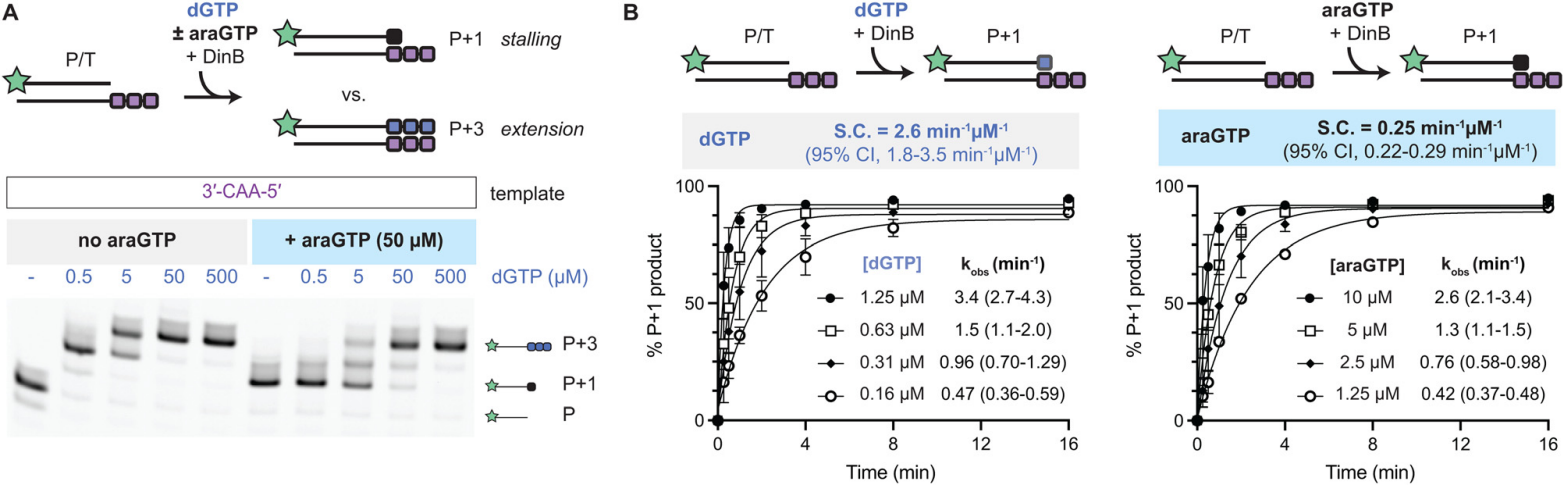
AraNTPs can effectively compete with dNTPs. (A) Assay schematic (top) and gel (bottom) for competition of dGTP and araGTP incorporation using a multi-incorporation template, with quantification provided in Fig. S9. (B) Plotted is the product formation as a function of time at various concentrations of dGTP (left) or araGTP (right) using the single-nucleotide incorporation template. The derived observed rates were used to determine the specificity constant (S.C.) for dGTP and araGTP. Each point is from 2–3 replicates per condition, with error bars showing standard deviation. Derived kinetic values are given as mean and 95% confidence interval (Fig. S5).

An important question for establishing araNTPs as potential DinB inhibitors is whether they can compete effectively with natural dNTPs. To explore competition, we focused our analysis on evaluating araGTP *versus* dGTP given prior work that has established some tolerance for nucleobase modifications with 8-oxo-dGTP and our observation of minimal extension after araGTP addition. We used a template containing a 3′-CCC-5′ overhang and evaluated extension by increasing concentrations of dGTP (0.5 μM to 500 μM) in the absence or presence of araGTP (50 μM) (Fig. 4(A)). In the absence of araGTP, extension proceeds to P + 2 with 0.5 μM dGTP and to the P + 3 product at 5 μM dGTP and above. However, in the presence of 50 μM araGTP, we instead observed the stalled P + 1 product as the dominant product with up to 5 μM dGTP (42% P + 1 product). Under equimolar conditions (50 μM araGTP/50 μM dGTP), the P + 1 and P + 2 truncated products remained detectable (>25%), yet were largely absent without araGTP (<10%). Thus, in direct competition experiments, we observe that araNTPs can compete with their natural dNTP counterparts for DinB-dependent incorporation *in vitro*.

To further support our competition-based experiment, we aimed to more rigorously establish the kinetic parameters governing araGTP *versus* dGTP incorporation. We returned to the single-incorporation template (3′-CAA-5′ overhang) and assayed under pre-steady state conditions with serial time points. Guided by prior work that has established a *K*_M_ for dGTP in the high μM range,^30^ we selected a range of low NTP concentrations that would allow us to accurately compare the two substrates under conditions where the rate is proportional to substrate concentration. As anticipated, each time course fits to a single exponential with observed rates increasing as a function of NTP concentration (Fig. 4(B)). The *k*_obs_ value at each concentration can be used to derive a specificity constant for dGTP and araGTP by linear regression. Imputing across the substrate range, we found the specificity constant for araGTP to be only 10-fold less than that of dGTP (Fig. S5). This result aligns well with our observation of stalling on the multi-incorporation template when using 50 μM araGTP and 5 μM dGTP. Taken together, our kinetic analysis substantiates that araNTPs can compete effectively as substrates with natural nucleotides, making these NTPs potentially viable antagonists of DinB.

### Combining sugar and nucleobase modifications leads to unique activity

**Fig. 5:**
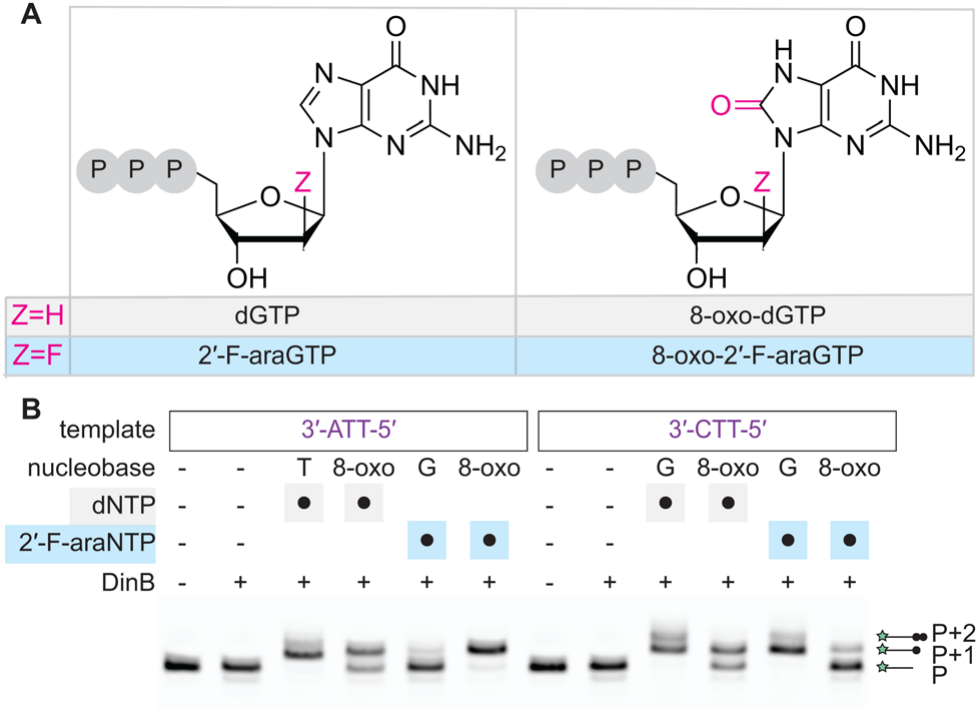
Nucleobase and sugar modifications can be combined to promote specific misincorporation. (A) Shown are the structures of dGTP, 2′-F-araGTP, and their 8-oxoguanine analogs. (B) Assay schematic (50 nM primer/template, 250 nM DinB, 1 mM nNTP) and results for incorporation of analogs, demonstrating efficient and preferential incorporation of 8-oxo-2′-F-araGTP opposite A over C. Quantification is provided in the Fig. S10.

Given the established tolerance of DinB for nucleobase modifications and our new insights into sugar-modified NTPs, we last explored whether base and sugar modifications could be considered in combination. As arabinosyl-8-oxoguanine triphosphate was synthetically inaccessible, potentially due to juxtaposition of the reactive 8-oxo moiety with the 2′-*S*-OH of the sugar, we instead evaluated 8-oxo-2′-F-araGTP, along with 8-oxo-dGTP and 2′-F-araGTP for comparison (Fig. 5(A)). In single-nucleotide extension assays, we employed either a template containing a 3′-ATT-5′ that would report on misincorporation of GTP analogs *via* Hoogsteen base pairing or a 3′-CTT-5′ overhang for cognate base pairing. We used each dNTP/nNTP at 1 mM, where cognate extension could be observed along with misincorporation, as evidenced from the generation of P + 1 and some P + 2 products using the 3′-ATT-5′ template with dTTP and the 3′-CTT-5′ template with dGTP (Fig. 5(B)). As previously established, we observed that DinB can insert 8-oxo-dGTP opposite either A or C to yield the P + 1 product. With the native guanine nucleobase, we observed that 2′-F-araGTP is preferentially inserted opposite its cognate C. Strikingly, with the 8-oxoguanine version, we instead find inversion of this preference with preferential misincorporation of 8-oxo-2′-F-araGTP opposite A and only limited incorporation opposite C.

To assess the impact of the Hoogsteen base pairing on extension, we turned to more complex substrates where multiple nucleotide additions could take place (Fig. S6). In these experiments, templates featured either a 3′-ACC-5′ or 3′-CAA-5′ overhang. We observed similar results with the addition of dTTP, dGTP, and 2′-F-araGTP, which can insert at cognate, matched sites. Interestingly, 8-oxo-dGTP extends to generate a P + 2 product with the 3′-CAA-5′ substrate, but only a P + 1 product with 3′-ACC-5′, indicating that the Hoogsteen pairing is less efficiently extended. With 8-oxo-2′-F-araGTP, we again observe the preferential misincorporation opposite A, with minimal extension, highlighting a degree of specificity in stalling for this analog. Together, these experiments suggest that combined nucleobase and sugar modifications lead to unique patterns of incorporation and extension, showing the potential to leverage pairings of nucleotide modifications to target specific polymerases.

## Experimental

### Cloning, expression, and purification of DinB-His

*E. coli dinB* was amplified from the BL21(DE3) strain and cloned into the pET41 plasmid with a C-terminal His tag. pET41-DinB-His plasmid was transformed into Rosetta 2(DE3) pLysS cells. A 2 L expression culture was inoculated with 20 mL of an overnight culture and grown at 37 °C to an OD of ∼0.6. IPTG was added to 1 mM and the culture was grown at 37 °C for 1 hour after induction. Cells were harvested by centrifugation at 2800 rcf for 20 minutes at 4 °C and resuspended in 20 mL of DinB Lysis Buffer (50 mM Tris–HCl pH 7.5, 300 mM NaCl, 10% glycerol, 30 mM imidazole) containing a Mini cOmplete EDTA-free protease inhibitor cocktail tablet (Sigma). Lysozyme (250 μg mL^−1^) and benzonase (2.5 units per mL) were added, and the cells were lysed by sonication. Lysate was clarified by centrifugation at 25 000 rcf for 20 minutes at 4 °C. The resulting supernatant was incubated for 1 hour at 4 °C with 3 mL HisPur Cobalt Resin (Thermo Scientific) pre-equilibrated with DinB Lysis Buffer. The protein-bound resin was washed with DinB Lysis Buffer and eluted with DinB Elution Buffer (30 mM Tris–HCl pH 7.5, 300 mM NaCl, 10% glycerol, 180 mM imidazole). DinB-His was dialyzed into DinB Storage Buffer (35 mM Tris–HCl pH 7.5, 60 mM NaCl, 25% glycerol, and 5 mM TCEP). Purity was assessed by SDS-PAGE (Fig. S1D), and the most prominent bands were characterized using LC–MS/MS (Fig. S1E) performed by Wistar Institute's Proteomics Core, demonstrating high purity and no evidence of prominent co-purification of accessory factors. Concentration was determined by Qubit Protein Kit (Thermo). DinB-His was aliquoted and stored at −80 °C.

### Nucleotide triphosphates

Nucleotide triphosphates were purchased from various sources, as documented in Table S1. 8-oxo-2′-F-araGTP was obtained from a custom synthesis by TriLink Biotechnologies, with the product confirmed by mass spectrometry and ^1^H and ^31^P NMR. All nucleotide triphosphates were stored at −20 °C.

### Primer/templates

*In vitro* primer extension assays were performed using pre-annealed primer/template (P/T) mixes. See Fig. S2 for P/T oligonucleotides (IDT). The annealing reaction contained 1 μM FAM-labeled primer and 2 μM template in 1X NEB rCutSmart buffer (50 mM potassium acetate, 20 mM Tris-acetate, 10 mM magnesium acetate, 100 μg mL^−1^ recombinant albumin at pH 7.9). Annealing was completed by heating to 95 °C for 5 minutes followed by a linear ramp to room temperature over 1 hour. Annealed product was aliquoted and stored at −20 °C until use.

### Primer extension assays

Primer extension assays for qualitative single or multiple incorporations contained 50 nM P/T mix and 250 nM DinB-His with 50 μM of the appropriate dNTP and/or nNTP in 1X NEB rCutSmart Buffer, unless otherwise specified. Reactions were incubated at 37 °C for 1 hour. Reactions were quenched with 2X Formamide Loading Dye (95% Formamide, 20 mM EDTA, and 0.04% Bromophenol Blue). Samples were denatured for 5 minutes at 95 °C and held at 55 °C until loading onto a pre-heated 20% polyacrylamide gel containing 7 M urea. Samples were run for approximately 30 minutes or 1 hour at 40 W and imaged using the FAM setting of a Typhoon (GE). Notably, with 30 minutes separation on gel, there is less distinction between P and P + 1 bands. However, with 1 hour separation on the gel, a minor shadow is more apparent above the P band, intrinsic to the primer alone (Fig. S2E). With either time selected, appropriate background correction was performed to quantify the extension products as detailed below.

For the assays aimed at examining subsequent extension after initial incorporation of araGTP, the reaction conditions were similar to those for single incorporation (described above); however, after the one-hour reaction at 37 °C, dTTP was spiked in to a final concentration of 500 μM along with additional DinB-His (to maintain 250 nM total final concentration) and rCutSmart Buffer (to maintain 1X final concentration).

Similar assays were performed for qualitative competition assays. The P/T mix and DinB-His concentrations as well as buffer conditions were as above, however, the concentration of araGTP was 50 μM and the corresponding dGTP concentrations were 0, 0.5, 5, 50, or 500 μM.

Assays with 8-oxo-dGTP, 2′-F-araGTP, and 8-oxo-2′-F-araGTP were performed as described above; however, these nNTPs were studied at a final concentration of 1 mM.

### Pre-steady state kinetic assays

Pre-steady state kinetic assays were performed with DinB-His at 250 nM. The P/T concentration was 50 nM. A range of dGTP (0.16, 0.31, 0.63, and 1.25 μM) and araGTP (1.25, 2.5, 5, and 10 μM) concentrations were tested based on ability to capture early primer extension. Assays were performed at 25 °C and timepoints were taken at *t* = 0, 0.25, 0.5, 1, 2, 4, 8, and 16 minutes. Samples were denatured and analyzed using 20% polyacrylamide gels containing 7 M urea with imaging as described above. Gels were quantified using ImageJ (NIH). Quantified data (Table S2) were fit with single-association non-linear curve-fitting ([P + 1] = [P] (1 – e^−*k*_obs_*t*^)) in Prism (GraphPad). The individual rates from each concentration (*k*_obs_) were then fit to obtain the slope of the early phase of the Michaelis–Menten curve (*k*_obs_ = (S.C.) [NTP] + *b*), yielding the determined specificity constant (S.C., equivalent to *k*_pol_/*K*_D_) for dGTP and araGTP with DinB.

### Quantification of product formation

Gels were quantified using ImageJ. Each species was quantified and corrected for background contribution and the values were converted to % by accounting for all relevant species. For assays with multiple incorporations, the %P values for +NTP conditions were corrected by subtracting the %(P − 1) band for the no NTP control and the %(P + 1)+(P + 2) values for +NTP conditions were corrected by subtracting the %(P + 1) band for the no NTP control. All uncropped gels are provided in the Fig. S7–S10, along with the quantification for each gel.

## Conclusions

In this work, we have demonstrated that the error-prone, TLS DNA polymerase DinB can tolerate a subset of sugar modifications in the incoming nucleotide triphosphate. We identified araNTPs as analogs that are incorporated proficiently and can effectively compete with natural dNTPs, resulting in stalling after incorporation. These results suggest that although DinB has evolved to exclude rNTPs, its more open and less flexible active site makes it permissive to 2′-*S* modifications, including those in araNTPs and 2′-F-araNTPs, raising the prospect that other 2′-*S* modifications could be explored to further enforce specificity. Interestingly, we also found that tolerance for base modifications could be further exploited in combination with sugar modifications, with the example of 8-oxo-2′-F-araGTP preferentially misincorporating opposite A rather than C. We posit that this selectivity likely results from the presence of the 2′-*S*-fluoro substituent enforcing a preference for the syn over the anti-conformation, which then favors misincorporation opposite A.^31^ The fact that nucleobase and sugar alterations can be combined to give distinctive patterns offers opportunities for finding distinctive pairings of nucleotide analogs and polymerases.

DNA damaging antibiotics are key inducers of the bacterial SOS response, during which DinB is significantly upregulated.^2,5^ Prior work has demonstrated that, in the presence of DinB overexpression, increasing 8-oxo-dGTP levels or blocking its repair after incorporation can result in selective lethality.^19^ This precedent raises the possibility that TLS polymerase promiscuity could be harnessed as an antibacterial approach, with our findings supporting the extension of this approach to sugar-modified analogs. Challenges to such an approach include the need to generate nNTPs in cells,^32^ which could require existing bacterial machinery to activate the associated nucleosides, or nucleotide prodrug strategies, as with tenofovir.^33,34^ Furthermore, navigating selectivity for bacterial *versus* mammalian TLS polymerases would be another potential requirement, given the precedent of incorporation or extension of cytarabine (araC) by some mammalian polymerases and 2′-F-araNTP by some A- and B-family DNA polymerases.^35^ Taken together, our results highlight the mechanistic value of using nNTPs as probes of enzyme promiscuity and suggest pathways that could expand the antibacterial arsenal to include nucleosides targeting the effectors involved in promoting antibiotic tolerance and resistance.

## Author contributions

Conceptualization – CMH, JMK, LCW, RMK; data curation, methodology, validation, and formal analysis – lead by CMH, with contributions from JMK, MBC, JBP, CEL, LCW; project administration and funding acquisition – RMK; writing and visualization – RMK, CMH; review and editing – all authors.

## Conflicts of interest

There are no conflicts to declare.

## Supplementary Material

CB-OLF-D5CB00100E-s001

## Data Availability

The data supporting this article have been included as part of the SI, including uncropped gels and quantification in Fig. S7–S10. Supplementary information: Fig. S1–S10 and Tables S1, S2. See DOI: https://doi.org/10.1039/d5cb00100e
