# Supplementary material for: The bacterial stress response polymerase DinB tolerates sugar modifications and preferentially incorporates arabinosyl nucleotides
Source: RSC Chem Biol. 2025 Sep 1;6(10):1650–6. doi: 10.1039/d5cb00100e (PMC12415624; doi:10.1039/d5cb00100e)
Supplement: CB-006-D5CB00100E-s001 [file CB-006-D5CB00100E-s001.pdf]

Supplementary Information (SI) for RSC Chemical Biology.
This journal is © The Royal Society of Chemistry 2025

SUPPLEMENTARY INFORMATION

The bacterial stress response polymerase DinB tolerates sugar modifications and preferentially incorporates arabinosyl nucleotides

Christina M. Hurley[a], Jeffrey M. Kubiak[b], Michael B. Cory[a], Jared B. Parker[c], Christian E. Loo[a], Laura C. Wang[c], and Rahul M. Kohli[c,d,*]

[a] Graduate Group in Biochemistry, Biophysics, and Chemical Biology, Perelman School of Medicine, University of Pennsylvania, Philadelphia, PA, USA

[b] Graduate Group in Cell and Molecular Biology, Perelman School of Medicine, University of Pennsylvania, Philadelphia, PA, USA

[c] Department of Medicine, Perelman School of Medicine, University of Pennsylvania, Philadelphia, PA, USA

[d] Department of Biochemistry and Biophysics, Perelman School of Medicine, University of Pennsylvania, Philadelphia, PA, USA

*Correspondence to: Rahul M. Kohli (rkohli@pennmedicine.upenn.edu)

Table S1. Nucleotide triphosphates examined and commercial sources………………… …2
Table S2. Kinetic measurements of dGTP and araGTP incorporation…………………. …3

Figure S1. DinB structure and purification……………………………………………….. …4
Figure S2. Primer/template pairs and control experiments…………………………….... …6
Figure S3. Single-incorporation primer extension assay quantification………………. …7
Figure S4. Incorporation and extension assays…………………………………….…... …8
Figure S5. Kinetic evaluation of dGTP and araGTP incorporation……………………… …9
Figure S6. Combined nucleobase and sugar modifications with alternative templates……..……………………………………………………………………… 10
Figure S7. Uncropped gel scans and quantification for single-incorporation primer extension assays…………………………………………………………………………….. …11
Figure S8. Uncropped gel scans and quantification of extension……………………… …13
Figure S9. Uncropped gel scans for competition assays with quantification of assay featuring a multiple incorporation template……………………………………………. …15
Figure S10. Uncropped gel scans and quantification for assays with 8-oxo-G analogs……………………..…………………………………………………..………… …18

Table S1. Nucleotide triphosphates examined and commercial sources. Vendor and product numbers are listed for each analog studied. Gray boxes indicate analogs that were either unavailable or not evaluated.

	Nucleobase					
	A	C	G	T	U	8-oxo-G
3′-deoxyribosyl-NTP (dNTP)	Promega, U120A	Promega, U122A	Promega, U121A	Promega, U123A		TriLink, N-2034-BGF01A
ribosyl-NTP (rNTP)	NEB, N0451A	NEB, N0454A	NEB, N0452A		NEB, N0453A	
2′,3′-dideoxyribosyl-NTP (ddNTP)	TriLink, N-4001-1	Trilnk, N-4005-1	AAT Bioquest, 17210	Roche, 11326900 TriLink N-4004-1		
3′-amino-dideoxyribosyl-NTP (3'-NH_2)	TriLink, N4010-T1LA01A			TriLink, N-4013-071002		
3′-azido-dideoxyribosyl-NTP (3'-N_3)	TriLink, N-4007-011008			TriLink, N-4009-111208B		
3′-methoxy-ribosyl-NTP (3'-OCH_3)	TriLink, N-1056-101204				TriLink, N-1059-040604	
acycloNTP	NEB, N0461A	NEB, N0462A	NEB, N0463A	NEB, N0464A		
2′-fluorodeoxyribosyl-NTP (2′-F-dNTP)	TriLink, N-1007-T1NH01A	TriLink, N-1008-013008	TriLink, N-1009082603	TriLink, N-1055-120701		
arabinosyl-NTP (araNTP)	TriLink, N-1048-031901	TriLink, N-1033-030602	TriLink, N-1100 T1-BGX01A		TriLink, N-1034-061109	
2′-fluoroarabinosyl-NTP (2'-F-araNTP)			Jena Bioscience, NU-10507-G-10			Trilink (Custom Synthesis)

Table S2. Kinetic measurements of dGTP and araGTP incorporation. Time course replicate product formation quantification for Figure 4B and derived k_{obs} values for determination of specificity constant for Figure S5.

Time (min)	1.25 μM dGTP		0.63 μM dGTP		0.31 μM dGTP		0.16 μM dGTP		
0.00	0.0	0.0	0.0	0.0	0.0	0.0	0.0	0.0	0.0
0.27	47.4	67.7	24.9	40.4	23.7	26.2	17.0	24.7	7.2
0.50	67.5	79.7	38.7	57.3	31.5	44.1	22.4	29.0	18.4
1.0	83.2	87.7	60.7	78.9	46.5	63.3	32.3	38.9	37.9
2.0	89.8	90.8	79.4	86.4	64.5	80.2	45.9	55.9	58.1
4.0	92.5	91.8	88.2	88.7	80.0	86.1	61.2	72.4	76.0
8.0	94.2	93.8	92.2	92.2	88.5	89.2	78.1	83.2	85.3
16	94.8	94.4	92.8	93.0	90.3	92.3	87.6	89.2	89.6

Time (min)	10 μM araGTP			5 μM araGTP			2.5 μM araGTP		1.25 μM araGTP	
0.00	0.0	0.0	0.0	0.0	0.0	0.0	0.0	0.0	0.0	0.0
0.25		41.0	61.4		21.2	36.0	9.2	16.2	13.5	7.1
0.50	52.8	64.6	79.7	40.6	42.1	52.9	24.0	37.0	19.6	12.8
1.0	76.0	80.5	89.0	59.9	63.5	76.0	39.3	58.5	34.1	33.1
2.0	87.2	89.4	91.1	76.8	80.8	83.3	63.6	76.5	51.8	52.2
4.0	92.3	91.4	91.9	88.8	89.6	88.5	81.5	85.9	69.9	72.2
8.0	93.9	93.8	92.9	92.6	93.0	91.1	90.1	90.5	84.9	84.4
16	95.5	95.2	94.0	94.7	94.9	92.0	92.6	92.4	92.0	89.7

[dGTP] μM	k_{obs} (min^{-1})		
	mean	lower limit	upper limit
1.25	3.40	2.70	4.30
0.63	1.48	1.10	1.96
0.31	0.96	0.70	1.29
0.16	0.47	0.36	0.59

[araGTP] μM	k_{obs} (min^{-1})		
	mean	lower limit	upper limit
10.0	2.64	2.08	3.37
5.0	1.29	1.08	1.53
2.5	0.76	0.58	0.98
1.3	0.42	0.37	0.48

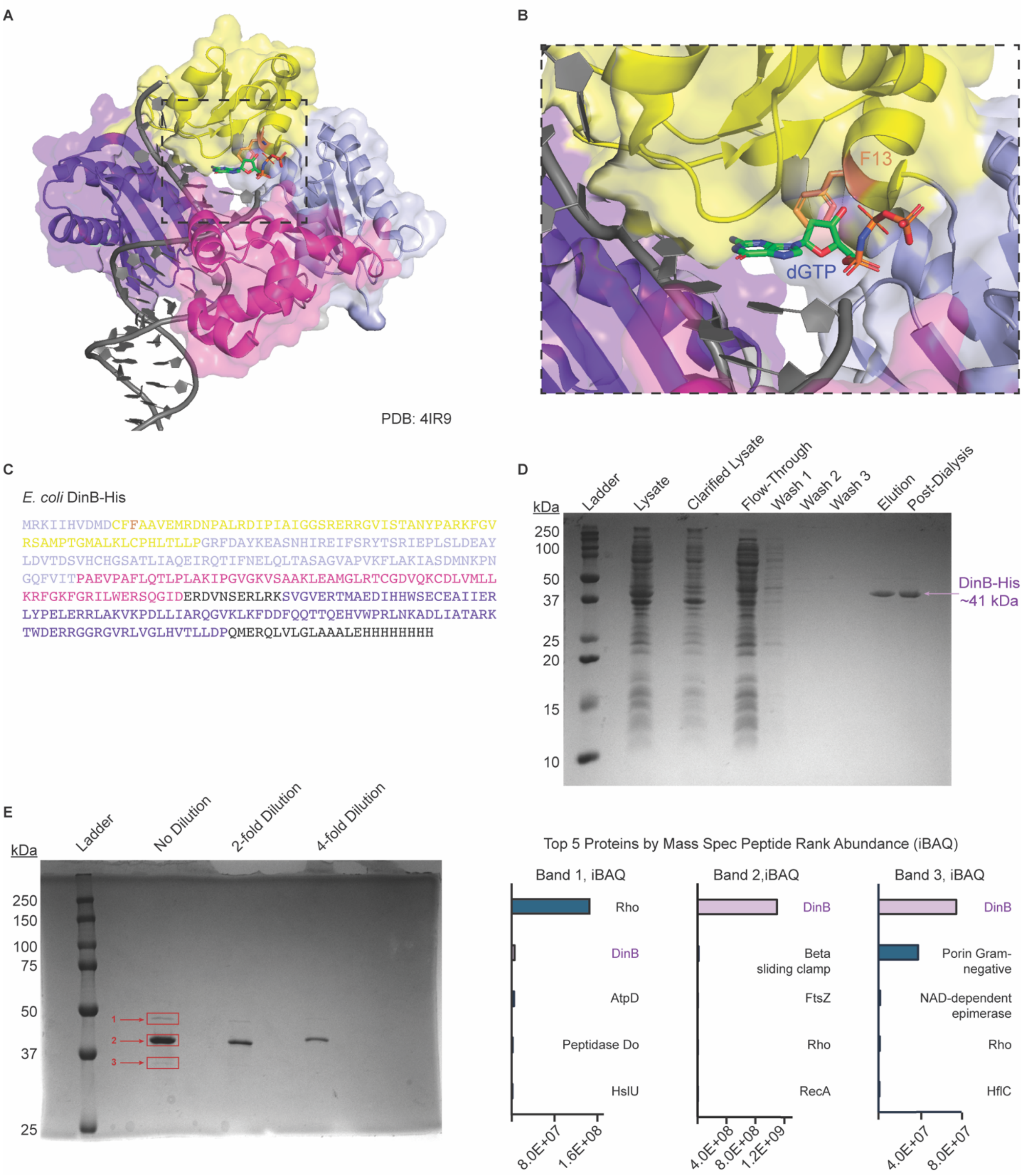

Figure S1. DinB structure and purification. A) The DNA- and dGTP-bound structure of DinB is shown (PDB 4IR9) highlighting the palm (light purple), fingers (yellow), thumb (pink), and PAD (dark purple) domains with the active site region indicated by the dotted box. B) Zoom-in of active site shows the steric gate residue, F13 (orange), involved in discrimination of rNTPs. C) The sequence of the expressed DinB-His protein, with domain coloring corresponding to that in (A). D) Uncropped SDS-PAGE gel tracking overexpression and purification of DinB-His is

shown, with purified DinB-His at right. E) Uncropped SDS-PAGE gel showing bands excised for LC-MS/MS analysis. Three bands selected for analysis are noted. Each was excised, reduced with TCEP, alkylated with iodoacetamide, and digested with trypsin. Digested samples were analyzed with 40 min-LC gradient and the peptide fragments analyzed on an Orbitap Astral Mass Spectrometer (Thermo). The resulting data was searched against the UniProt *E. coli* (DE3) database (UP000002032) and converted to protein abundance as shown. Plotted is the Intensity-Based Absolute Quantification (iBAQ) for each protein and band.

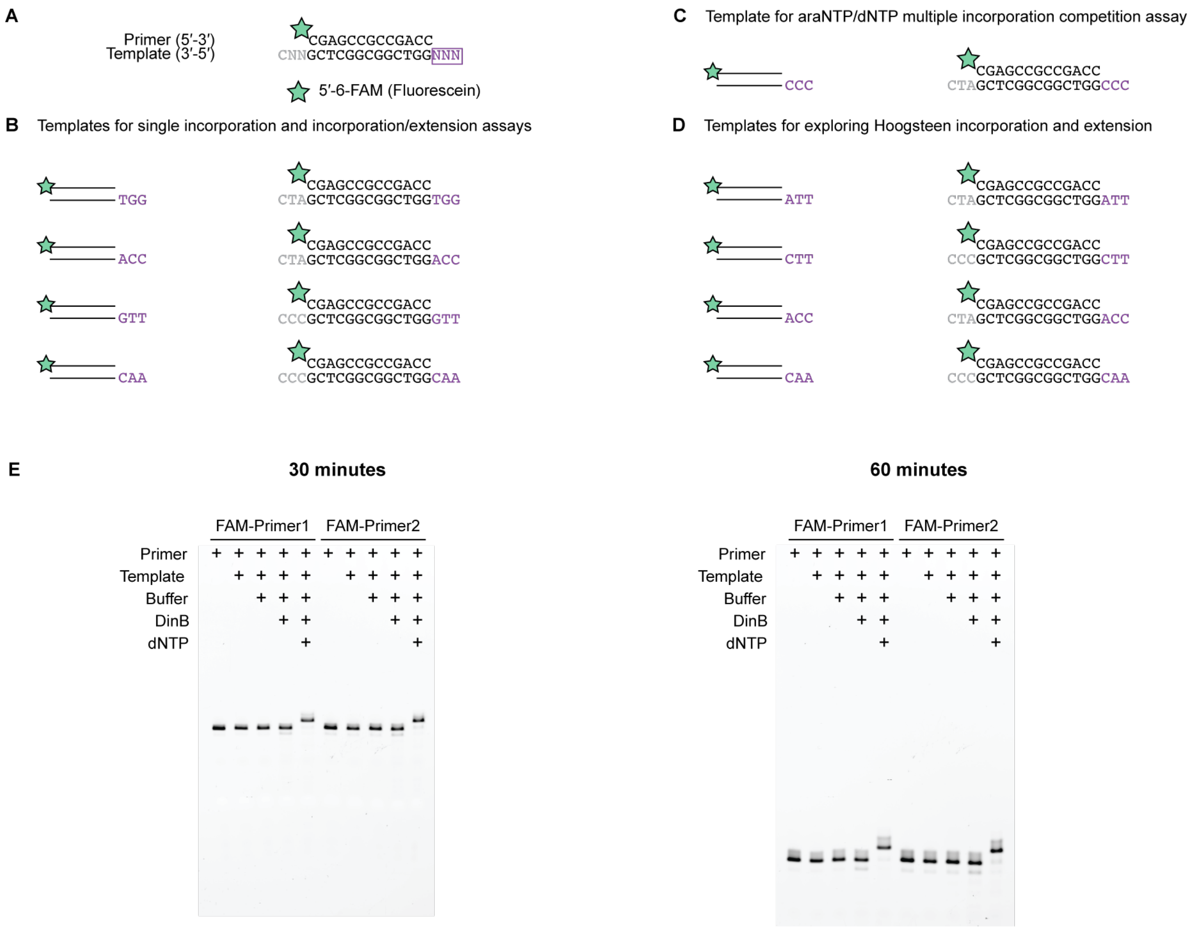

Figure S2. Primer/template pairs and control experiments. A) Schematic shown for the primer (P) and templates (T) evaluated. The primer contains a 5′-6-fluorescein label for visualization of primer and extension products. Shown are the templates for B) single incorporation and incorporation/extension assays, C) multiple incorporation assay for competition, and D) incorporation and extension assay for evaluating canonical and Hoogsteen base pairing. E) Uncropped gels of control experiments showing the primer alone (P), with additions of template (P/T), buffer, DinB, and dNTPs. At left is the gel separation performed for 30 min, while at right is gel separation performed for 1 hour. Within each gel are shown two separate syntheses of the FAM-labeled with FAM-Primer1 resulting from HPLC purification alone, with FAM-Primer2 from dual HPLC purification. While 30 min separation results in less distinction between the P and P+1 bands, at 1 hour there is a shadow above the P and P/T bands that can interfere with detection of some P+1 products. These findings are the same for both FAM-Primer1 and FAM-Primer2 highlighting that they are intrinsic to the primer and separation conditions. A trace P-1 truncation is detectable in the P and P/T alone, but enhanced in the presence of DinB, suggested trace exonuclease contamination, which was accounted for in quantifying extension reactions with multiple incorporations. Fig. 2, 3, 5, and S4A-B, use 30 min separation, while Fig. 4, S4C, S5, and S6 use 1 hour separation.

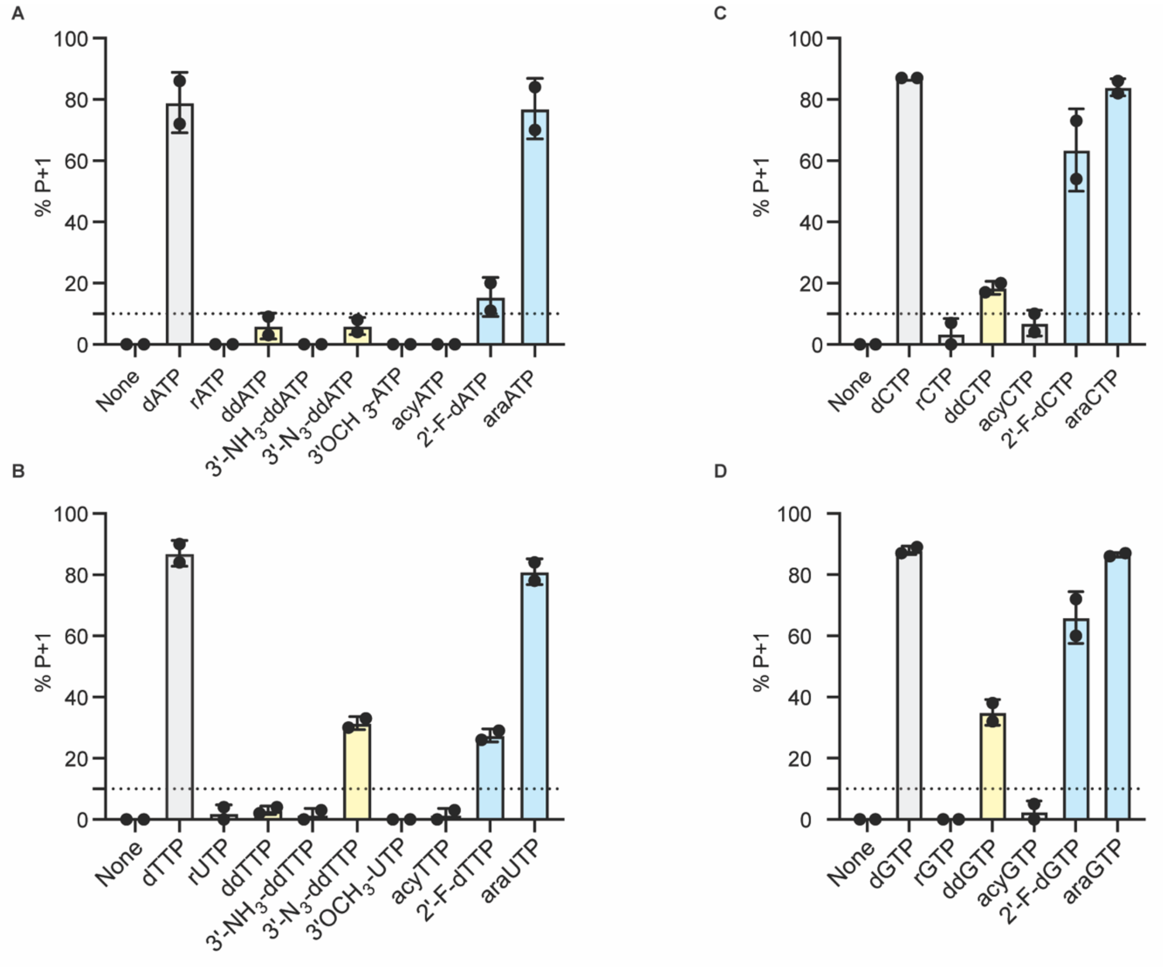

Figure S3. Single-incorporation primer extension assay quantification. Bar graphs showing % P+1 for each nNTP tested in Fig. 2. Individual data points are shown from duplicate analysis. Representative gels are shown in Fig. 2C-F with uncropped gels and resulting quantification in Fig. S7. Error bars are standard deviation from the mean. The dotted line at 10% shows the threshold for determining no efficient incorporation (<10%). A) nATPs. B) nTTP/nUTPs C) nCTPs D) nGTPs. Bars are shaded to match the color scheme in Fig. 2, with 3′-modified nNTPs and 2′-modified nNTPs highlighted in yellow and blue, respectively.

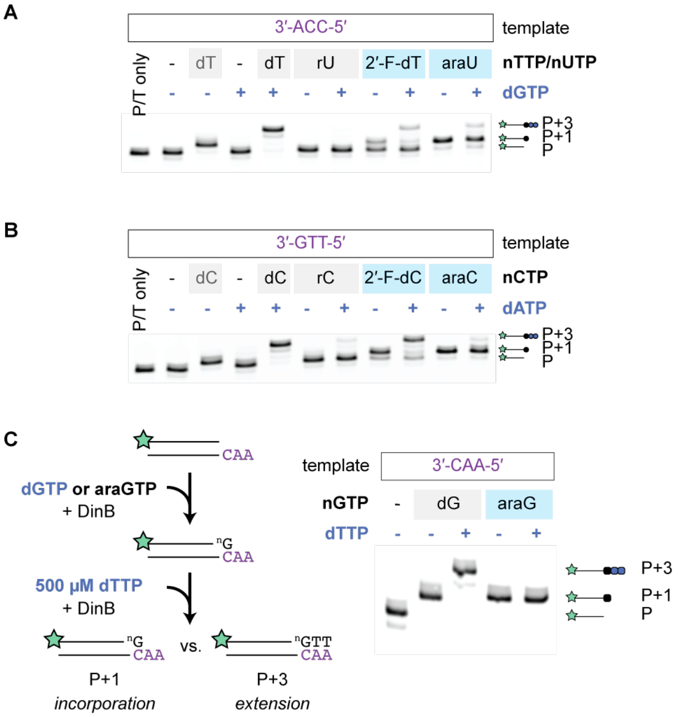

Figure S4. Incorporation and extension assays. Shown are the incorporation and extension assays with A) nTTP/UTP + dGTP and B) nCTP + dATP. C) Assay schematic (at left) and gel (at right) for evaluation of extension under driving conditions with incorporation (50 nM primer/template, 250 nM DinB, 50 µM dGTP/araGTP), followed by 500 µM dTTP. Uncropped gels and quantification are shown in Fig. S8.

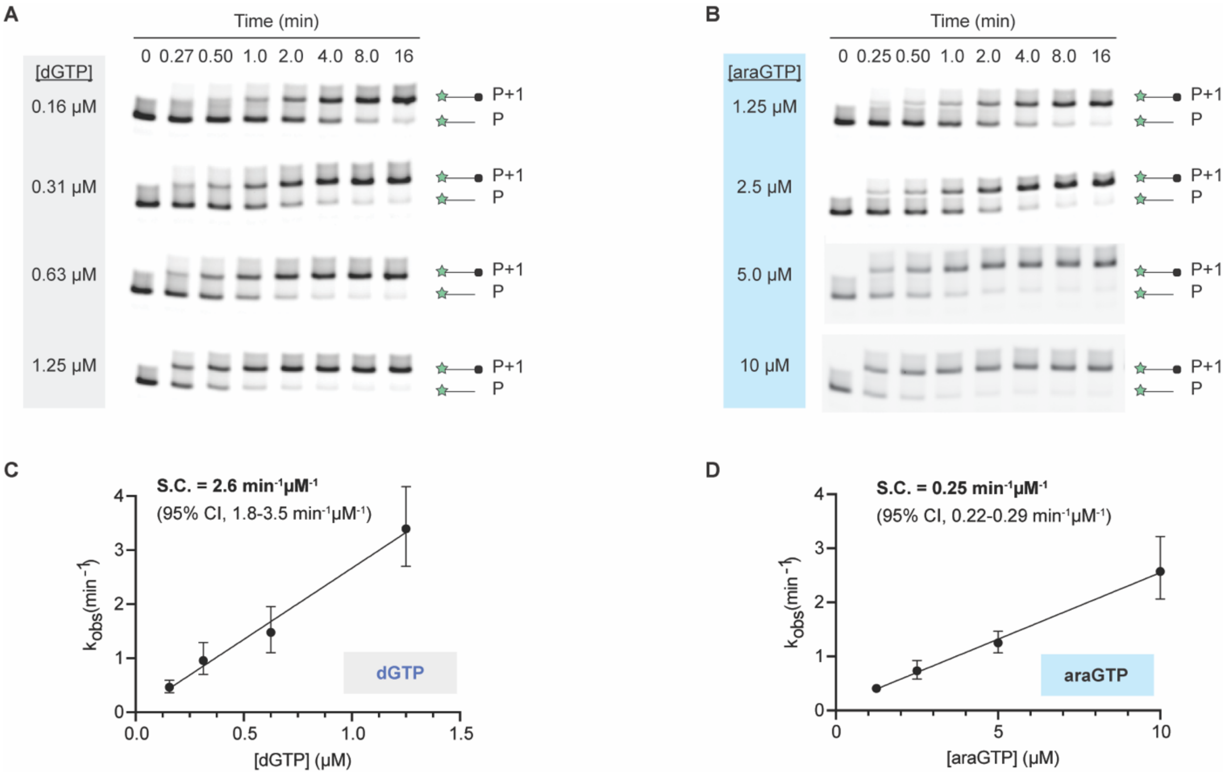

Figure S5. Kinetic evaluation of dGTP and araGTP incorporation. A) Shown are representative gels from a single incorporation assay using dGTP at various concentrations (0.15 – 1.25 μM) and time points (0 – 16 min). B) Shown are representative gels from a single incorporation assay using araGTP at various concentrations (1.25 – 10 μM) and time points (0 – 16 min). Uncropped gels are shown in Fig. S9. C) k_{obs} values obtained from analysis of individual substrate concentration conditions were used to obtain the specificity constant for dGTP, as reported in Figure 4B. D) k_{obs} values obtained from analysis of individual substrate concentration conditions were used to obtain the specificity constant for araGTP, as reported in Figure 4B.

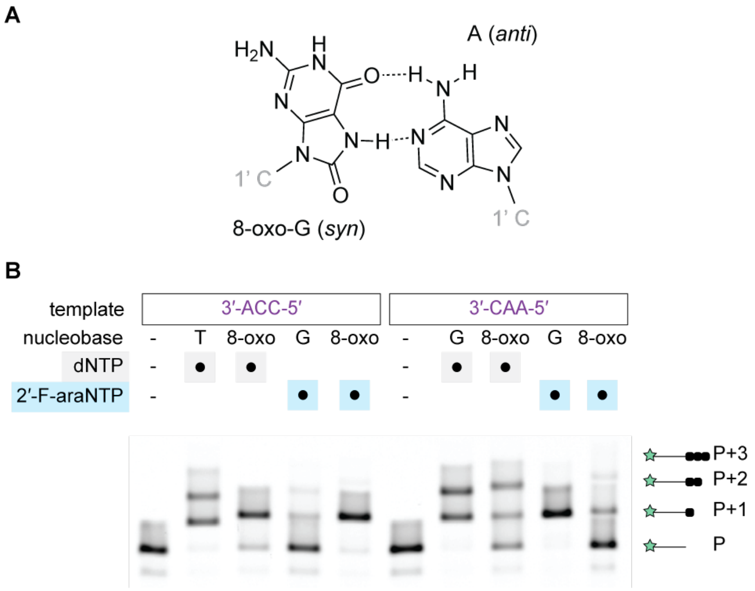

Figure S6. Combined nucleobase and sugar modifications with alternative templates. A) Schematic of 8-oxo-G Hoogsteen base pairing shown for reference. B) Incorporation of analogs using alternative templates that have 3′-ACC-5′ or 3′-CAA-5′ overhangs that could be permissive for multiple incorporations using combined cognate and Hoogsteen base pairing, demonstrating efficient and preferential incorporation of 8-oxo-2′-F-araGTP opposite A over C. Uncropped gels and quantification are shown in Fig. S10.

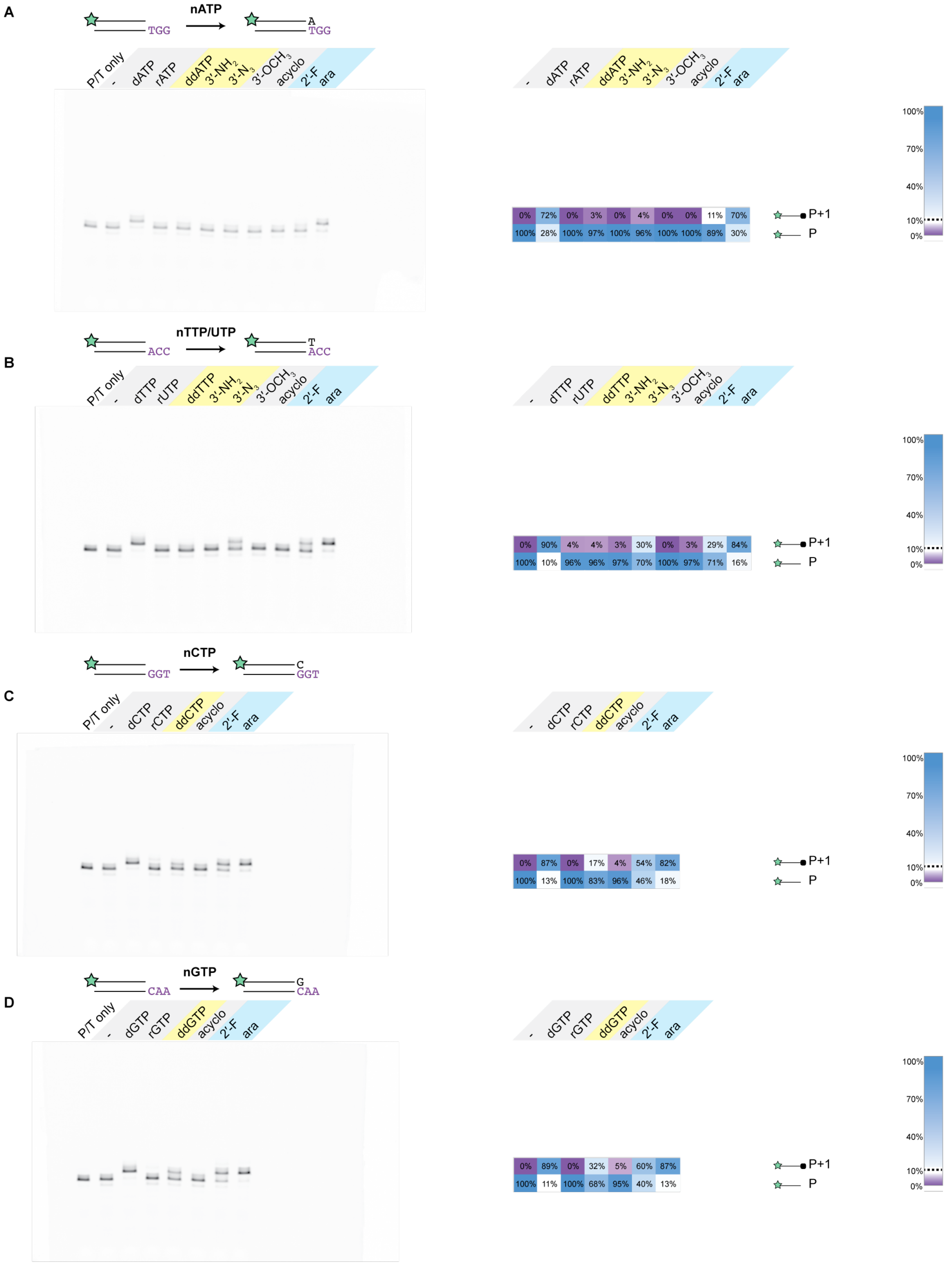

Figure S7. Uncropped gel scans and quantification for single-incorporation primer extension assays. Shown are the uncropped gel scans (left) and quantification (right) for the

experimental results shown in Fig. 2. A) Fig. 2C. B) Fig. 2D, C) Fig. 2E. D) Fig. 2F. For quantification, shades of purple indicate that the percent of P or P+1 <10% of the total, white indicates that the percent of P or P+1 is equal to 10% of the total, and shades of blue indicate that the percent of P or P+1 is >10% of the total. The quantified data from this analysis and a separate independent replicate were integrated in the data shown in Fig. S3.

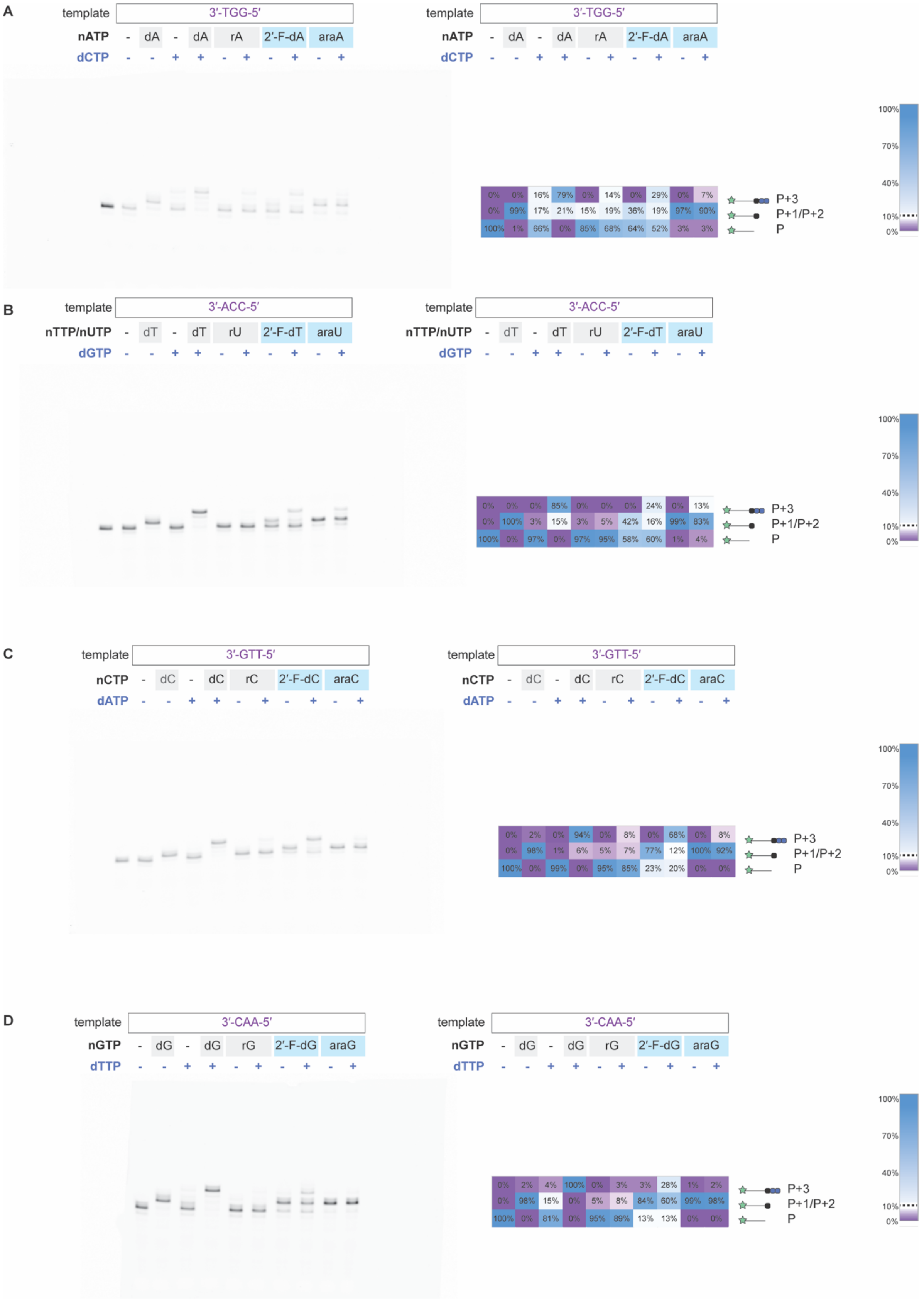

A
template 3′-TGG-5′
nATP - dA - dA rA 2′-F-dA araA
dCTP - - + + - + - + - +
0% 0% 16% 79% 0% 14% 0% 29% 0% 7%
0% 99% 17% 21% 15% 19% 36% 19% 97% 90%
100% 1% 66% 0% 85% 68% 64% 52% 3% 3%
P+3
P+1/P+2
P
B
template 3′-ACC-5′
nTTP/nUTP - dT - dT rU 2′-F-dT araU
dGTP - - + + - + - + - +
0% 0% 0% 85% 0% 0% 0% 24% 0% 13%
0% 100% 3% 15% 3% 5% 42% 16% 99% 83%
100% 0% 97% 0% 97% 95% 58% 60% 1% 4%
P+3
P+1/P+2
P
C
template 3′-GTT-5′
nCTP - dC - dC rC 2′-F-dC araC
dATP - - + + - + - + - +
0% 2% 0% 94% 0% 8% 0% 68% 0% 8%
0% 98% 1% 6% 5% 7% 77% 12% 100% 92%
100% 0% 99% 0% 95% 85% 23% 20% 0% 0%
P+3
P+1/P+2
P
D
template 3′-CAA-5′
nGTP - dG - dG rG 2′-F-dG araG
dTTP - - + + - + - + - +
0% 2% 4% 100% 0% 3% 3% 28% 1% 2%
0% 98% 15% 0% 5% 8% 84% 60% 99% 98%
100% 0% 81% 0% 95% 89% 13% 13% 0% 0%
P+3
P+1/P+2
P
100%
70%
40%
10%
0%

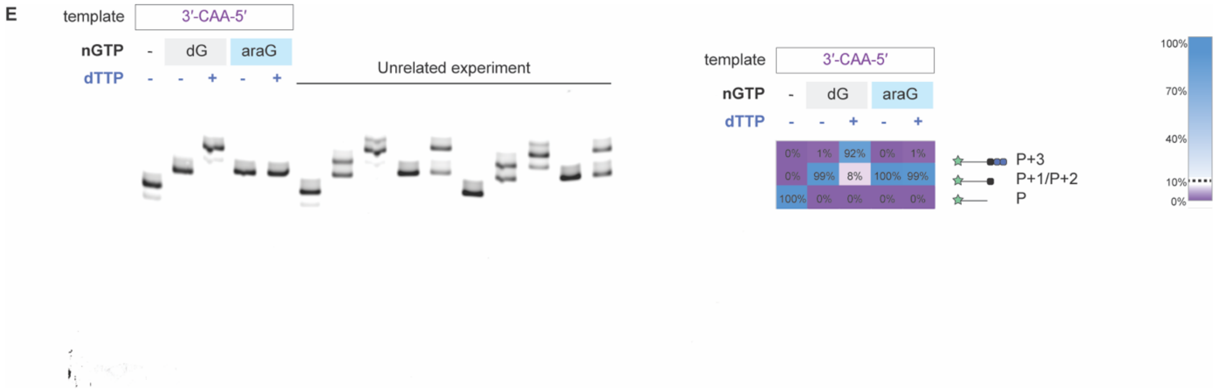

Figure S8. Uncropped gel scans and quantification of extension. Shown are the uncropped gel scans (left) and quantification (right) for the experimental results shown in Fig. 3 and Fig. S4. A) Fig. 3B. B) Fig. S4A, C) Fig. S4B. D) Fig. 3C. E) Fig. S4C. For quantification, shades of purple indicate that the percent of the given species <10% of the total, white indicates that the percent of the given species is equal to 10% of the total, and shades of blue indicate that the percent of the given species is >10% of the total.

A

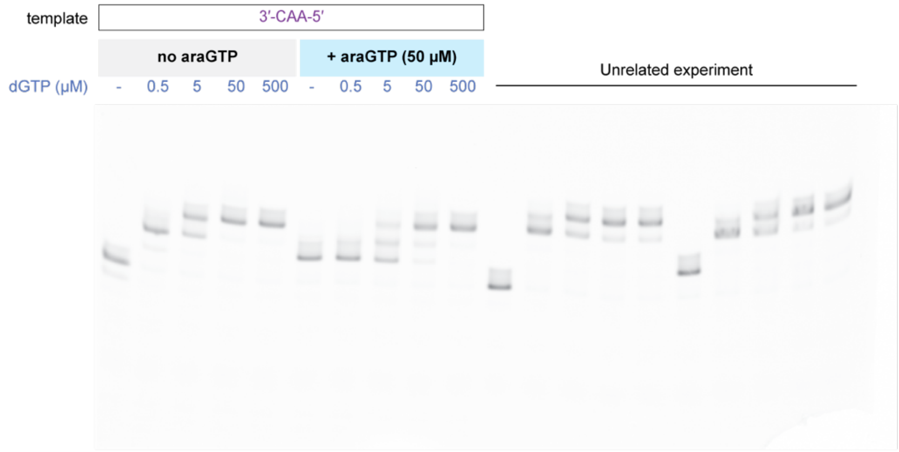

template	3′-CAA-5′										
	no araGTP					+ araGTP (50 µM)					
dGTP (µM)	-	0.5	5	50	500	-	0.5	5	50	500	
	0%	4%	58%	95%	92%	0%	1%	24%	74%	92%	P+3
	0%	80%	41%	5%	6%	8%	9%	34%	18%	7%	P+2
	0%	16%	2%	0%	2%	91%	90%	42%	9%	2%	P+1
	100%	0%	0%	0%	0%	0%	0%	0%	0%	0%	P

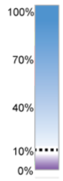

B

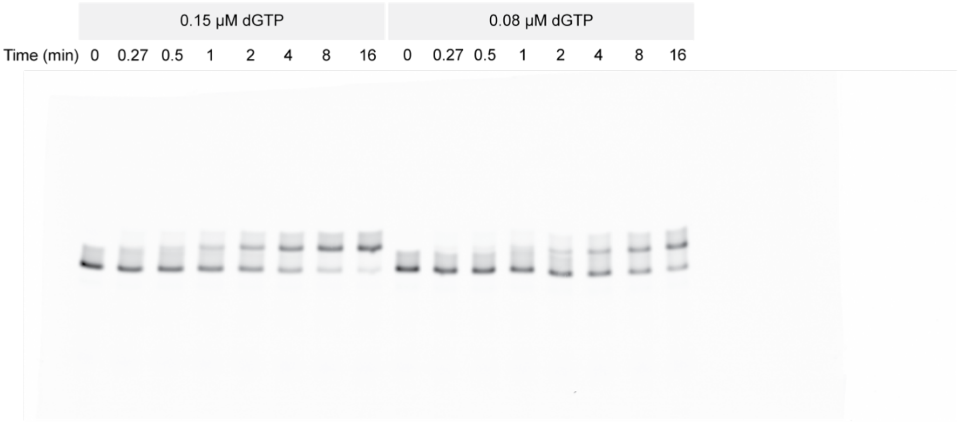

C

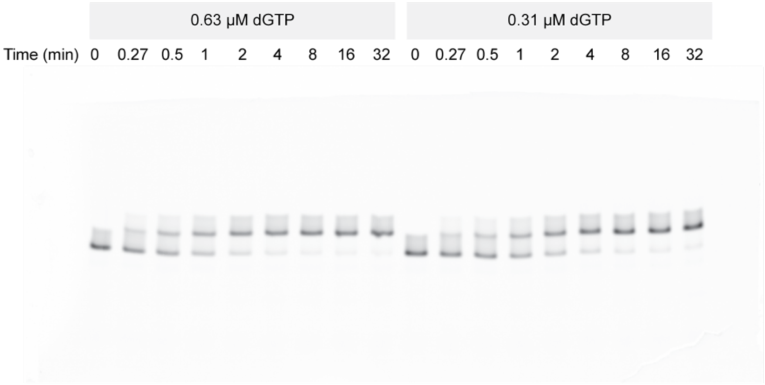

D

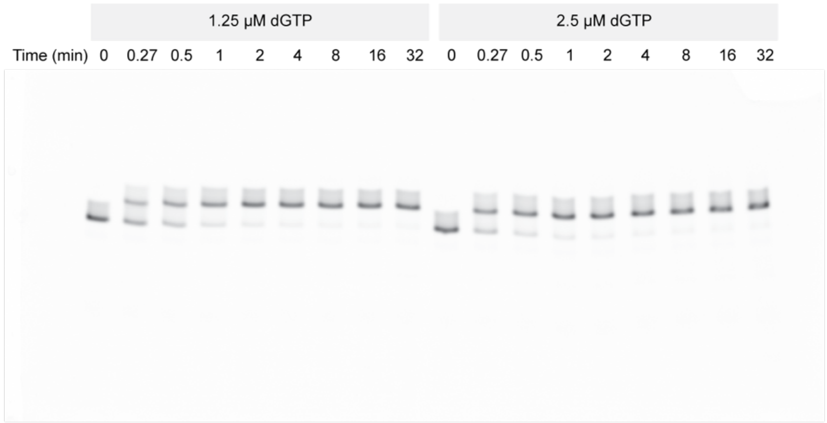

E

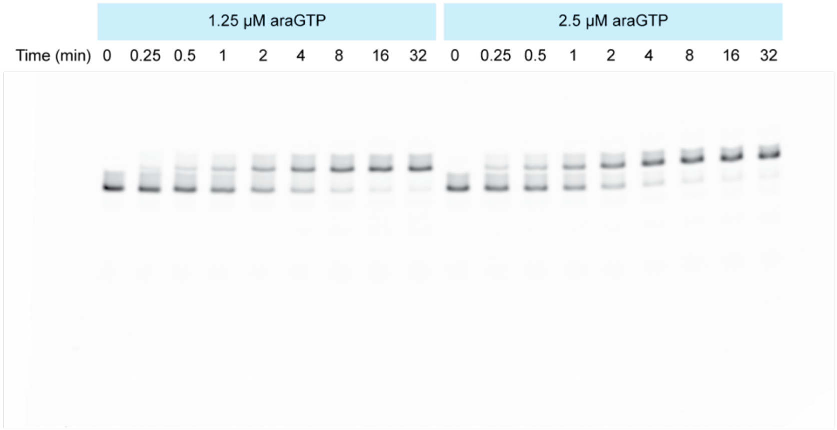

F

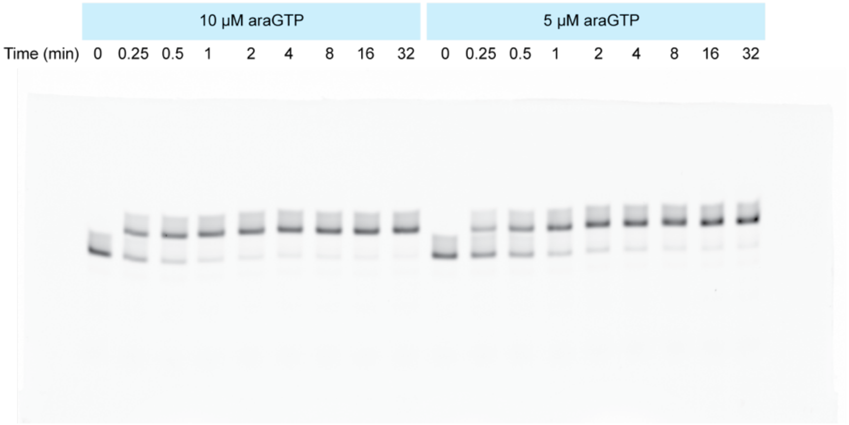

Figure S9. Uncropped gel scans for competition assays with quantification of assay featuring a multiple incorporation template. A) Shown is an uncropped gel scan (top) for Fig. 4A and quantification (bottom). For quantification, shades of purple indicate that the percent of the given species <10% of the total, white indicates that the percent of the given species is equal to 10% of the total, and shades of blue indicate that the percent of the given species is >10% of the total. B-D) Shown are uncropped gel scans for Fig. S5A. Note that 32 min timepoints were excluded due to reaction completion at earlier timepoints. Data for 0.08 and 2.5 μM dGTP are not discussed, as these concentrations were less informative given the timepoints

examined. E-F) Shown are uncropped gel scans for Fig. S5B. Note that 32 min timepoints were excluded from the analysis due to reaction completion at earlier timepoints.

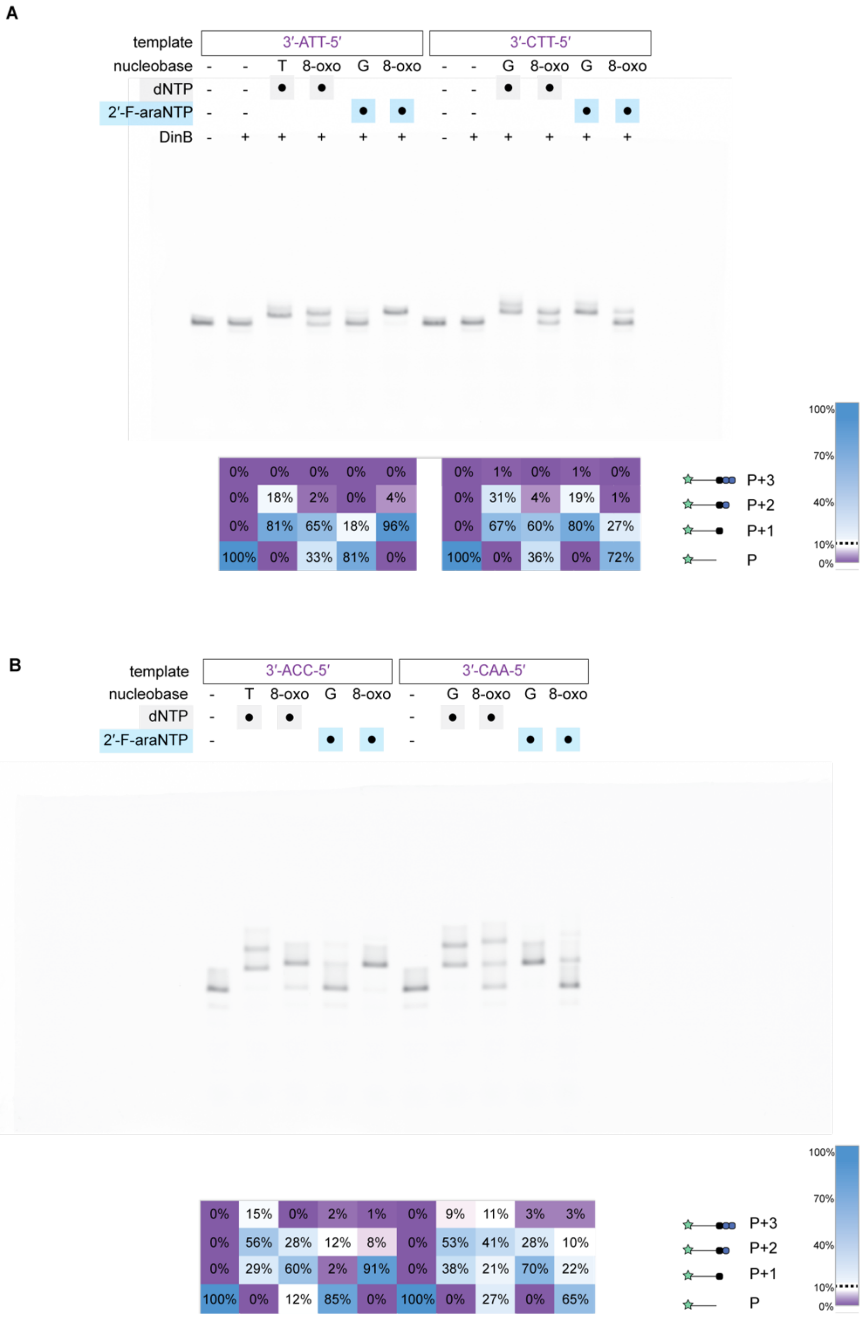

Figure S10. Uncropped gel scans and quantification for assays with 8-oxo-G analogs. Shown are the uncropped gel scans (top) and quantification (bottom) for the experimental results in A) Fig. 5B and B) Fig. S6B. For quantification, shades of purple indicate that the percent of the given species <10% of the total, white indicates that the percent of the given species is equal to 10% of the total, and shades of blue indicate that the percent of the given species is >10% of the total.
